# The relationship between consumption of nitrite or nitrate and risk of non-Hodgkin lymphoma

**DOI:** 10.1038/s41598-020-57453-5

**Published:** 2020-01-17

**Authors:** Mengxia Yu, Chenying Li, Chao Hu, Jingrui Jin, Shenxian Qian, Jie Jin

**Affiliations:** 1 Department of Hematology, The First Affiliated Hospital, College of Medicine, Zhejiang University, #79 Qingchun Road, Hangzhou, Zhejiang Province 310003 P.R. China; 2 Department of Hematology, Hangzhou First People’s Hospital, College of Medicine, Zhejiang University, #216 Huansha Road, Hangzhou, Zhejiang Province 310006 P.R. China; 3 Key Laboratory of Hematopoietic Malignancies, Diagnosis and Treatment, #17 Laozhedazhi Road, Hangzhou, Zhejiang Province 310009 P.R. China

**Keywords:** B-cell lymphoma, Risk factors

## Abstract

Epidemiologic studies of the relationship between nitrite or nitrate consumption and risk of non-Hodgkin lymphoma (NHL) remain controversial. The current meta-analysis aimed to reexamine the evidence and quantitatively evaluate that relationship. Manuscripts were retrieved from the Web of Science, Chinese National Knowledge Infrastructure and PubMed databases up to May 2019. From the studies included in the review, results were combined and presented as odds ratios (OR). To conduct a dose-response (DR) analysis, studies presenting risk estimates over a series of categories of exposure were selected. Our data indicate that the consumption of nitrite was linked to a significantly increased hazard of NHL (OR: 1.37; 95% CI: 1.14–1.65), rather than nitrate (OR: 1.02; 95% CI: 0.94–1.10). According to Egger’s and Begg’s tests (*P* > 0.05), there was no evidence of significant publication bias. Moreover, our DR analysis indicated that the risk of NHL grew by 26% for each additional microgram of nitrite consumed in the diet per day (OR: 1.26; 95% CI: 1.09–1.42). Through subset analysis of the nitrite studies, data from the high-quality studies indicated that consumption was positively associated with carcinogenicity, leading to NHL (OR: 1.44; 95% CI: 1.17–1.77) and positively correlated with the development of diffuse large B-cell lymphoma (OR: 1.55; 95% CI: 1.07–2.26), but not other NHL subtypes. In addition, the data suggested that females (OR: 1.50; 95% CI: 1.15–1.95) and high levels of nitrite intake (OR: 1.64; 95% CI: 1.28–2.09) had a higher risk of NHL. Our meta-analysis supports the hypothesis that nitrite intake, but not that of nitrate, raises the risk of developing NHL. In the future, better designed prospective research studies should be conducted to confirm our findings, clarify potential biological mechanisms and instruct clinicians about NHL prophylaxis.

## Introduction

Non-Hodgkin lymphoma (NHL) is a heterogeneous group of hematologic malignancies, developing from cells contained in the lymphoid tissue or from lymph glands. In recent decades, NHL has been categorized into more than 40 forms based on pathological and histological features by the World Health Organization^1^. According to the 2018 global cancer statistics, NHL ranks as the 8th most common carcinoma in males and 10th in females^2^. In the USA, it has been estimated that 74,680 new cases of NHL (41,730 males and 32,950 females) and 19,910 deaths (11,510 males and 8,400 females) occurred in 2018^3^. Despite the diagnostic and therapeutic progress in the recent decades^4–7^, the 5-year survival rate for all NHL forms combined is 72% (https://seer.cancer.gov/statfacts/html/nhl.html). It is well-known that NHL patients, particularly for high-risk subtypes (*e.g*. immunoblast lymphoma, Burkitt lymphoma and lymphoblastic lymphoma), continue to receive adverse prognoses. Hence, to increase precautions against and reduce the prevalence of NHL, further exploration of its risk and deeper understanding of its epidemiology are essential.

Nitrate and nitrite are crucial precursors of N-nitroso compounds (NOCs), a group of genotoxic composites that are highly carcinogenic and which act systemically^8^. In epidemiological studies, the potential relationship between nitrate or nitrite consumption and risk of development of tumors has been investigated. Positive associations have been detected in adult glioma (RR: 1.32; 95% CI: 1.01–1.71)^9^, thyroid neoplasm (RR: 2.05; 95% CI: 1.20–3.51)^10^, and gastric carcinoma (RR: 1.90; 95% CI: 1.30–2.70)^11^, *etc*. Moreover, Xie *et al*.^12^ and Song *et al*.^13^ further confirmed these correlations by conducting meta-analyses. During the past decades, the relationship between risk of NHL and nitrate or nitrite consumption have been investigated in several epidemiological publications, yet the results are contradictory. Some surveys failed to show positive or negative associations^14–21^, while others have revealed significant correlations^22–25^. When no specific trend can be ascertained from any individual investigation, combining a number of independent studies can reveal hidden associations through meta-analysis. Therefore, we conducted the present meta-analysis to discover latent connections between nitrate or nitrite consumption with the etiology of NHL, and so aimed to: (1) evaluate the epidemiological evidence about the relationship between nitrate or nitrite consumption from water or diet and risk of NHL; (2) consider a possible dose response (DR) relationship between nitrate or nitrite consumption and the risk of NHL; and (3) assess the quality of evidence and the statistical significance of the results.

## Results

### Study retrieval and research characteristics

**Figure 1:**
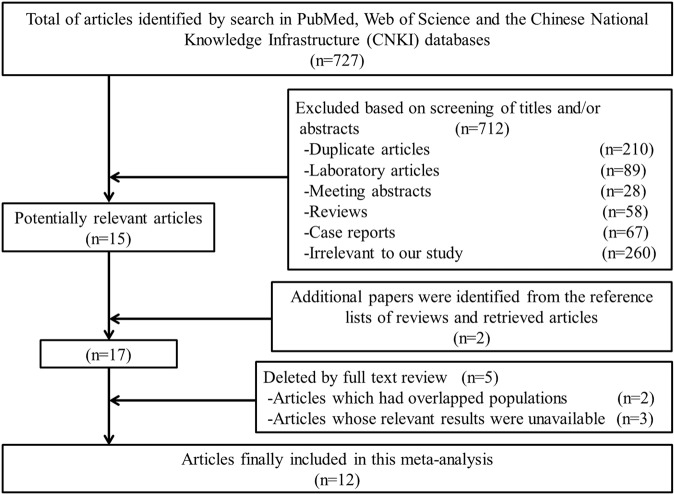
Flowchart describing the selection of studies included in the meta-analysis.

Figure 1 displays details of the process of identification of relevant studies and filtering of articles. In total, 727 relevant manuscripts were identified. After deletion of 210 duplicates, the titles and abstracts of the remaining 517 articles were inspected. Of these, 502 studies were removed for the following reasons: laboratory research study (n = 89), conference abstract (n = 28), review article (n = 58), case report (n = 67), irrelevant subject matter (n = 260). Thus, 15 papers apparently matched the inclusion criteria for further screening. In addition, two studies were identified from the reference lists of the retrieved and review articles. After reading the full texts, 8 case-control and 4 follow-up studies that reported the correlation between nitrate or nitrite consumption and risk of NHL published between 1996 and 2013 were selected^14–25^.

Table 1 displays the fundamental characteristics of the articles included in the review. The studies were conducted in three regions, as follow: Europe (n = 3; one from Italy, one from the United Kingdom and a third from Slovakia), North America (n = 8, all from the United States) and Asia (n = 1, from China). A total of 8,067 cases of NHL were included from all the studies. Four articles reported a significant relationship between nitrate or nitrite intake and the risk of NHL^22–25^. In each study included in the review, at least two different pathologists were involved in the diagnosis of NHL. Every NHL case was categorized by the Working Formulation criteria of the Lymphoma Study Group or using the World Health Organization (WHO) classification criteria^26,27^. The quality of each study was evaluated using the Newcastle-Ottawa Quality Assessment Scale (NOS). Scores ranged from 4 to 8 with a mean value of 6 (Supplementary Tables S1 and S2). Information on nitrate and nitrite intake was obtained by personal interview, telephone interview or mailed questionnaires.

**Table 1:** Principal characteristics of studies evaluating the association between nitrate or nitrite and risk of NHL.

Study	year	Country	Gender	Age	Study Design	Source of patients	Number of cases	Number of controls	Items	Source of nitrate and nitrite	Study Quality	Matching and Adjustments
Ward *et al*.	1996	United States	M/F	≥21	Case-control	Population-based	156	527	Nitrate	Diet^a^ and drinking water^b^	8	Race, gender, vital status, family history of cancer, vitamin C, carotenes, education, smoking and age
Ward *et al*.	2006	United States	M/F	20–74	Case-control	Population-based	1321	1057	Nitrate and nitrite	Diet and drinking water	7	Age, gender, center, race, education, caloric intake, study matching factors and gender
Aschebrook-Kilfoy *et al*.	2010	United States	F	21–84	Case-control	Population-based	594	710	Nitrate and nitrite	Diet	7	Age, family history of NHL, total daily energy intake, vitamin C intake, vitamin E intake, smoking and protein intake
Chiu *et al*.	2008	United States	M/F	≥21	Case-control	Population-based	147	1075	Nitrate and nitrite	Diet	8	Age, sex, type of respondent, family history of cancer, and body mass index
Cocco *et al*.	2003	Italy	M/F	≥10	Follow-up study	Population-based	737	NR	Nitrate	Drinking water	5	Gender, age, and population size
Law *et al*.	1999	United Kingdom	NR	0–79	Follow-up study	Population-based	2673	NR	Nitrate	Drinking water	4	Age, gender and population density
Freedman *et al*.	2000	United States	M	≥30	Case-control	Population-based	73	147	Nitrate	Drinking water	6	Age
Gulis *et al*.	2002	Slovak Republic	M/F	≥20	Follow-up study	Population-based	41	NR	Nitrate	Drinking water	5	NR
Aschebrook-Kilfoy *et al*.	2013	United States	M/F	20–75	Case-control	Population-based	335	469	Nitrate and nitrite	Diet	5	Sex, age, body mass index, education, family history of cancer, vitamin C and E intake, smoking, and total daily caloric intake
Chang *et al*.	2010	China	M/F	50–69	Case-control	Population-based	1716	1716	Nitrate	Drinking water	5	NR
Rhoades *et al*.	2013	United States	M/F	54–76	Case-control	Population-based	140	192	Nitrate	Drinking water	5	Age, BMI, smoking, education, family history of cancer, drinking water contaminants and sex
Weyer *et al*.	2001	United States	F	55–69	Follow-up study	Population-based	134	21977	Nitrate	Diet and drinking water	7	Age, vitamin C and E intake, physical activity, education, smoking, water source, total energy, dietary nitrate, fruits and vegetables, body mass index and waist-to-hip ratio

Abbreviations: M, male; F, female; NR, not reported; NHL, non-Hodgkin lymphoma; BMI, body mass index. aDiet: details were assessed from food frequency questionnaire. bDrinking water: details were assessed from official measurements.

### Risk assessment

**Figure 2:**
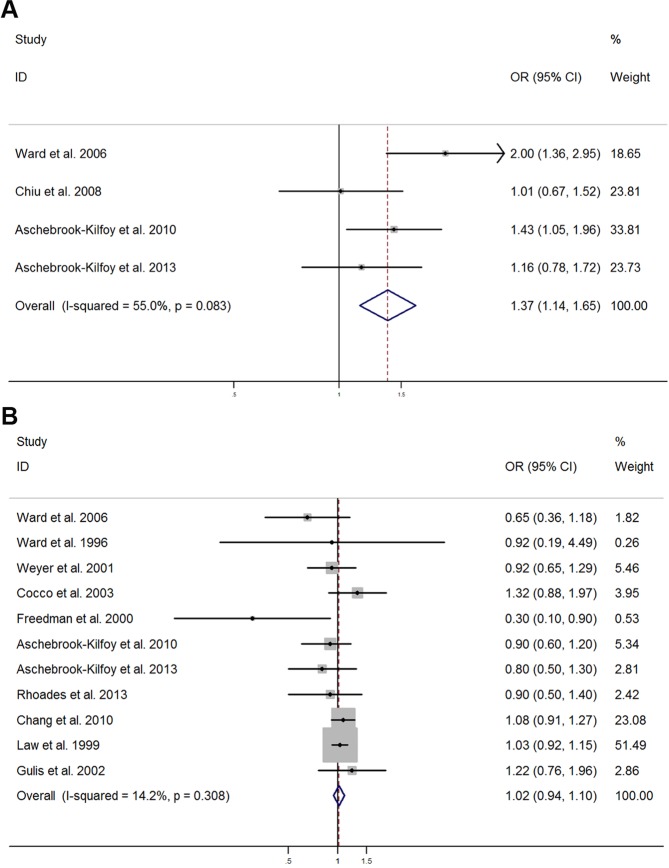
Forest plots illustrating risk estimates from studies included in the review on the relationships between nitrite (**A**) or nitrate intake (**B**) and the risk of NHL. The size of gray box is positively proportional to the weight assigned to each study, and horizontal lines represent the 95% confidence intervals.

**Figure 3:**
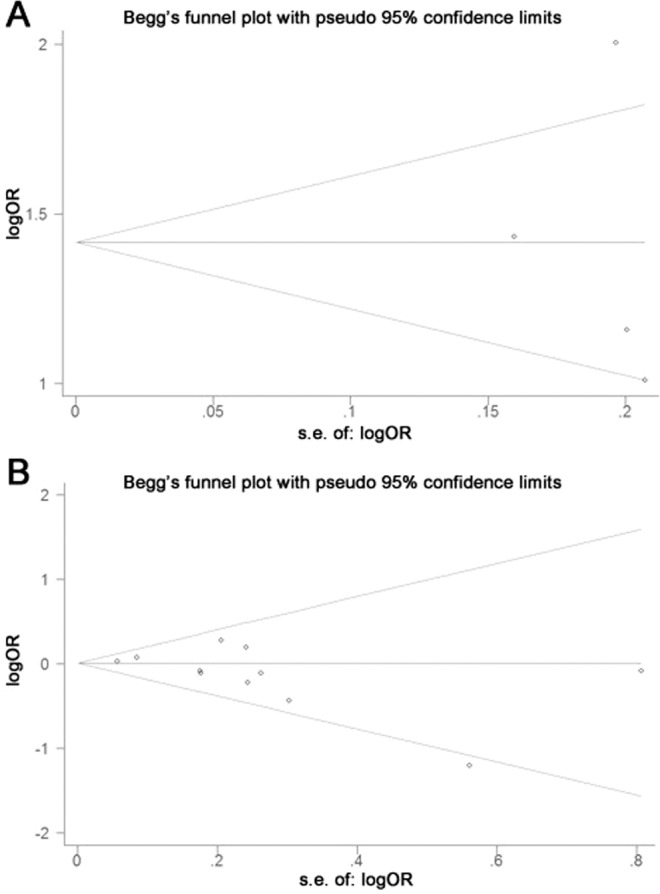
Funnel plots of: (**A**) nitrite or (**B**) nitrate intake (**B**) for risk of NHL.

**Figure 4:**
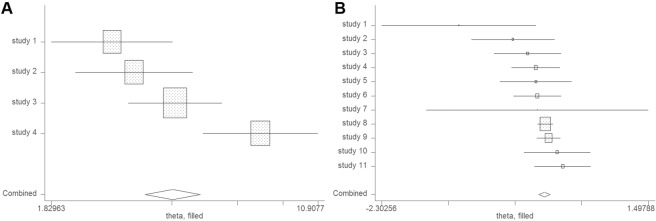
The trim-and-fill test did not identify possible missing studies for: (**A**) nitrite or (**B**) nitrate.

The relationship between nitrite or nitrate (high vs. low level consumption) and risk of NHL is illustrated in Fig. 2. The pooled ORs indicated that high levels of nitrite intake was linked to a significantly elevated risk of NHL (OR: 1.37; 95% CI: 1.14–1.65), yet no statistically significant heterogeneity was found (*I*^2^ = 55.0%, *P* = 0.083) (Fig. 2A). No evidence of publication bias were detected using an Egger’s or Begg’s test (*P* = 0.818 and 0.308, respectively) (Fig. 3A). Furthermore, no missing studies were identified using trim-and-fill analysis, further suggesting low publication bias (Fig. 4A). Conversely, no connection was found between the risk of NHL and high-levels of nitrate intake (OR: 1.02; 95% CI: 0.94–1.10) (Fig. 2B). No significant heterogeneity was found (*I*^2^ = 14.2%, *P* = 0.308), with both Egger’s and Begg’s tests indicating that no evidence of publication bias existed (*P* = 0.116 and 0.119, respectively) (Fig. 3B). Similarly, publication bias was not found by trim-and-fill analysis (Fig. 4B).

### Stratified analysis

Subset analyses were subsequently conducted, based on study design or quality, gender, source of nitrate, geographical region, NHL subtypes or levels of nitrite or nitrate (Tables 2 and 3). In subgroups divided by study quality, the high-quality studies suggested that nitrite intake affected tumorigenesis leading to NHL (OR: 1.44; 95% CI: 1.17–1.77), although a similar influence was not observed in the low-quality studies (OR: 1.16; 95% CI: 0.78–1.72). In the nitrate intake studies, no significant association was found in either the high (OR: 0.85; 95% CI: 0.66–1.04) or low (OR: 1.00; 95% CI: 0.91–1.09) quality studies. Gender was identified as a factor able to stratify the results in nitrite intake studies, with females exhibiting a significant positive association with NHL (OR: 1.50; 95% CI: 1.15–1.95) compared with males (OR: 0.84; 95% CI: 0.52–1.36). For nitrate consumption, the ORs (95% CI) were 1.00 (0.76–1.24) in the female group and 1.03 (0.61–1.46) in the male group. Where the risk factors concerned nitrite studies and NHL subtypes, a positive relationship was more evident in diffuse large B-cell lymphoma (DLBCL) (OR: 1.55; 95% CI: 1.07–2.26) compared with follicular lymphoma (FL) (OR: 1.29; 95% CI: 0.89–1.86). For nitrate studies, the ORs (95% CI) for DLBCL and FL were 0.86 (0.61–1.23) and 1.14 (0.80–1.63), respectively. When separately analyzed by study design of nitrate studies, no statistically significant relationship was observed either in follow-up studies (OR: 1.03; 95% CI: 0.93–1.14) or case-control studies (OR: 0.90; 95% CI: 0.77–1.02). For subgroup analysis based on geographical region, we found that nitrate intake was protective for NHL in North America (OR: 0.77; 95% CI: 0.62–0.92), but not in Asia (OR: 1.08; 95% CI: 0.90–1.26) or Europe (OR: 1.05; 95% CI: 0.94–1.16). Additionally, in subsets stratified by source of nitrates, no statistically significant difference was found between nitrates in the diet or drinking water (OR: 0.83; 95% CI: 0.68–1.01; OR: 0.96; 95% CI: 0.78–1.14, respectively). Finally, when analyzing by the levels of nitrite or nitrate, the high levels of nitrite in diet was positively associated with NHL (OR: 1.64; 95% CI: 1.28–2.09), but not for low-level group (OR: 1.08; 95% CI: 0.82–1.44). For nitrate studies, no significant connections were observed either in high-level group (OR: 1.05; 95% CI: 0.94–1.16) or low-level group (OR: 0.78; 95% CI: 0.30–1.26), respectively.

**Table 2:** Subgroup analyses of odds ratios for the relationship between nitrite intake and risk of NHL.

Variables	Number of studies	OR (95% CI)	Q-test for heterogeneity *P* value (*I*^2^ score)	*P* for interaction
Total	4	1.37 (1.14–1.65)	0.083 (55.0%)	
Gender				0.290
male	1	0.84 (0.52–1.36)	—	
female	2	1.50 (1.15–1.95)	0.608 (0.0%)	
Study quality				0.605
High	3	1.44 (1.17–1.77)	0.057 (65.2%)	
Low	1	1.16 (0.78–1.72)	—	
NHL subtype				0.800
DLBCL	2	1. 55 (1.07–2.26)	0.085 (66.3%)	
FL	2	1.29 (0.89–1.86)	0.045 (75.1%)	
Study design				—
Case-control study	4	1.37 (1.14–1.65)	0.083 (55.0%)	
Geographical area				—
United States	4	1.37 (1.14–1.65)	0.083 (55.0%)	
Source of nitrite				—
Diet	4	1.37 (1.14–1.65)	0.083 (55.0%)	
Levels in diet				0.170
High	2	1.64 (1.28–2.09)	0.186 (42.9%)	
Low	2	1.08 (0.82–1.44)	0.635 (0.0%)	

Abbreviations: NHL, non-Hodgkin lymphoma; DLBCL, diffuse large B-cell lymphoma; FL, follicular lymphoma.

**Table 3:** Subgroup analyses of odds ratios for the relationship between nitrate intake and risk of NHL.

Variables	Number of studies	OR (95% CI)	Q-test for heterogeneity *P* value (*I*^2^ score)	*P* for interaction
Total	11	1.02 (0.94–1.10)	0.308 (14.2%)	
Gender				0.767
male	5	1.03 (0.61–1.46)	0.333 (12.6%)	
female	6	1.00 (0.76–1.24)	0.722 (0.0%)	
Study quality				0.188
High	4	0.85 (0.66–1.04)	0.751 (0.0%)	
Low	7	1.00 (0.91–1.09)	0.015 (62.0%)	
NHL subtype				0.462
DLBCL	2	0.86 (0.61–1.23)	0.334 (0.0%)	
FL	2	1.14 (0.80–1.63)	0.141 (53.8%)	
Study design				0.988
Case-control study	7	0.90 (0.77–1.02)	0.028 (57.6%)	
Follow-up study	4	1.03 (0.93–1.14)	0.589 (0.0%)	
Geographical area				0.119
North America	7	0.77 (0.62–0.92)	0.272 (20.6%)	
Europe	3	1.05 (0.94–1.16)	0.505 (0.0%)	
China	1	1.08 (0.90–1.26)	—	
Source of nitrate				0.080
Diet	5	0.83 (0.68–1.01)	0.028 (63.1%)	
Water	9	0.96 (0.78–1.14)	0.019 (56.4%)	
Levels in the water				0.599
High	3	1.05 (0.94–1.16)	0.505 (0.0%)	
Low	3	0.78 (0.30–1.26)	0.002 (83.6%)	

Abbreviations: NHL, non-Hodgkin lymphoma; DLBCL, diffuse large B-cell lymphoma; FL, follicular lymphoma; NR, not reported.

### Dose-response (DR) analysis

**Figure 5:**
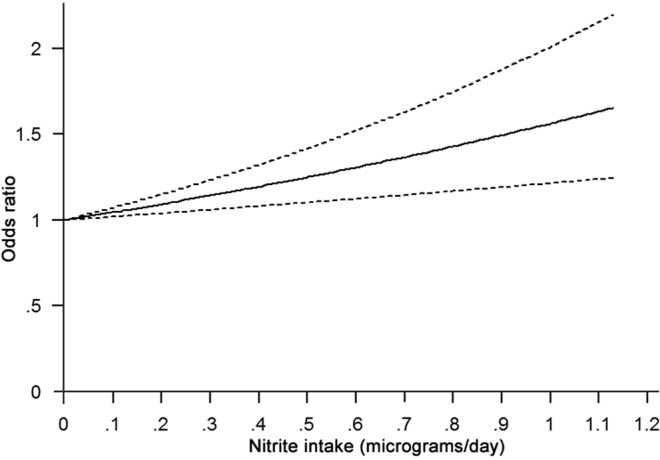
Odds ratio for NHL against dose of nitrite intake based on the results of the dose-response meta-analysis. Solid line represents estimated odds ratios, while the dotted lines represent 95% confidence intervals.

Because a heightened risk of NHL caused by the intake of nitrites, but not nitrates, was found, a DR analysis was further conducted to assess the dose-response interrelationship for the risk of NHL and nitrite consumption. A 26% greater risk of NHL was linked to an increase of 1 microgram of nitrite per day (OR: 1.26; 95% CI: 1.09–1.42) (Fig. 5).

### Heterogeneity appraisal

**Figure 6:**
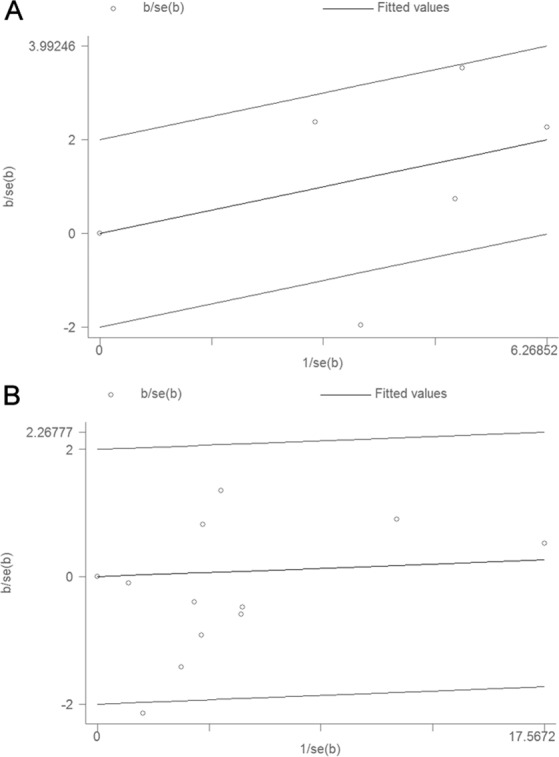
Galbraith plot analysis used to evaluate heterogeneity for: (**A**) nitrite and (**B**) nitrate studies.

In order to assess heterogeneity in the studies of this meta-analysis, the *I*^2^ statistic and Q test were utilized. We found large heterogeneity across the studies of nitrite (*P* = 0.083, *I*^2^ = 55.0%), but not studies of nitrate (*P* = 0.308, *I*^2^ = 14.2%) (Fig. 2). As shown in Fig. 6A for nitrite studies, we found that the major source of heterogeneity originated from a study exploring t(14;18)-negative NHL as displayed in the Galbraith plot. After precluding it, the heterogeneity decreased dramatically (*P* = 0.161, *I*^2^ = 41.8%). Furthermore, the overall association was more robust (OR: 1.55; 95% CI: 1.27–1.88). As displayed in Table 2, no factors could be identified as being the latent origin of heterogeneity in the nitrite studies when assessed by meta-regression analysis. For the nitrate studies, heterogeneity principally originated from the study of Freedman *et al*., as can be seen from the Galbraith plot (Fig. 6B). After excluding this study, which reported a considerable negative effect of nitrate intake for risk of NHL, but with low quality data (6), the heterogeneity disappeared (*I*^2^ = 0.0%, *P* = 0.588). The pooled OR remained not significant (1.00; 95% CI: 0.92–1.09). As shown in Table 3, no factors could be identified as being the potential origin of heterogeneity in the nitrate studies according to the meta-regression analysis.

### Sensitivity analysis

The influence of each study on overall estimate of risk was measured by repeating the meta-analysis after omitting each study in turn so as to conduct an analysis of sensitivity. As shown in Supplementary Fig. S1, no one item influenced the pooled OR for risk of NHL due to nitrate or nitrite consumption. The four study-specific ORs for nitrite intake and risk of NHL varied from a high of 1.54 (1.27–1.88) to a low of 1.23 (1.00–1.51) after removal of the t(14;18)-negative NHL study published by Chiu *et al*.^24^ and the research by Ward *et al*.^25^, respectively. For nitrate intake and risk of NHL, the ORs of the eleven studies varied from a high of 1.02 (0.95–1.11) to a low of 1.00 (0.91–1.09) after omitting the studies of Ward *et al*.^25^ and Chang *et al*.^20^, respectively.

## Discussion

Globally, nitrates and nitrites are present within many typical diets. For example, in processed meats (bacon, sausages, hot dogs, ham, *etc*.), nitrites and nitrates may be adjuncts that maintain the color of meat products and reduce microbial spoilage. However, excessive intake of processed meat is associated with an elevated risk of carcinomas, including NHL^28^. In recent years, the association between risk of NHL and nitrate or nitrite intake have been explored in a number of epidemiological studies. However, the relationship remains uncertain. Therefore, all available studies that have explored these relationships were integrated into this meta-analysis in an attempt to clarify these possible associations.

The meta-analysis summarized the outcomes of four follow-up and eight case-control studies, including four studies on nitrite consumption comprising a total of 1,542 cases and eleven on nitrate intake comprising a total of 7,920 cases. As far as can be ascertained, this is the first time an assessment of the relationship between the incidence of NHL and nitrate or nitrite consumption from water or diet has been performed by meta-analysis. The results indicate that a high nitrite intake from diet resulted in a 37% increased risk of NHL. Furthermore, DR analysis demonstrated that an additional consumption of 1 microgram/day of nitrite was linked to a 26% increased risk of NHL. However, this analysis suggests that there was no significant correlation between the risk of NHL and nitrate consumption (OR: 1.02; 95% CI: 0.94–1.10). In summary, the results suggest that nitrite intake, but not nitrate, is an important factor in the occurrence of NHL and that increased nitrite intake appears to raise the risk of NHL in a dose-dependent manner.

It is well-known that nitrates are abundant within the environment. Furthermore, nitrates participate in the nitrogen cycle which is essential for life. The International Agency for Research on Cancer (IARC) declared, as early as 2010, that there was no conclusive evidence that nitrates were carcinogens^29^. In fact, this meta-analysis was in agreement with previous studies, the results of which indicate that nitrate consumption was not linked to increased risk of NHL. Moreover, in subtype analysis where study quality or design, geographical area, source of nitrate, NHL subtypes, levels of nitrate in water and gender were considered, the risk of NHL was not increased as a result of nitrate consumption. Indeed, in representative exposure models, several studies have provided strong evidence that nitrate cannot be a carcinogen for humans or animals^30,31^. Notably, Palli *et al*., Rogers *et al*. and Ward *et al*. separately demonstrated that high nitrate consumption protected against esophageal cancer, gastric cancer and renal cell carcinoma^32–34^. However, high nitrate intake was deemed to be from the consumption of vegetables, which are also rich in various anti-cancer substances (including fiber, vitamin C, vitamin E and other anti-oxidants^13^.

In 1979, Newberne *et al*. observed that nitrite intake promoted lymphomas in rats in a dose-dependent manner^35^. In agreement with this study, our results indicated a significant positive relationship was found between NHL and high levels of nitrite intake (OR: 1.64; 95% CI: 1.28–2.09), but not for low levels of nitrite intake (OR: 1.08; 95% CI: 0.82–1.44). However, the mechanisms by which nitrites could influence the pathogenesis of NHL require clarification. A number of potential mechanisms could be responsible for nitrites being a risk factor for NHL. Previous studies have reported that nitrites can be converted into NOCs in the stomachs and intestines of both humans and animals through nitrosation with amides and amines. It is generally believed that NOCs are among the strongest known carcinogens, including *N*-nitrosodiethylamine (NDEA), *N*-nitrosopiperidine (NPIP), *N*-nitrosopyrollidine (NPYR), *N*-nitrosodimethylamine (NDMA) and *N*-Nitrosodibutylamine (NDBA), *etc* ^30,31,36^. They are considered genotoxic procarcinogens associated with driving tumorigenesis in a variety of tissues in more than 40 species, even senior primates^37,38^. In agreement with previously published reports, Storer *et al*.^39^ found that NDEA was able to effectively increase the frequency of malignant lymphoma in Eμ-pim-1 transgenic mice. Additionally, through the interplay of superoxides and NO production nitrites are able to form peroxynitrites which have powerful oxidative capability that causes damage to DNA through cellular oxidation, closely associated with carcinogenesis^40,41^. *In vitro*, Ustyugova *et al*.^42^ demonstrated that nitrites could inhibit Th1 cytokine formation, including interferon-γ, tumor necrosis factor-β and interleukin-2. Interestingly, Saberi *et al*. confirmed that such a change in concentration of these cytokines, at least partly, contributes to the increased risk of developing NHL^43^. In our study, a significant positive relationship was showed between the risk of NHL and nitrite consumption for female (OR: 1.50; 95% CI: 1.15–1.95), but not for male (OR: 0.84; 95% CI: 0.52–1.36). The different hormone and cytokine levels in female and male might be a possible reason for raising the risk of NHL. A greater number of basic studies are urgently required in order to clarify the biological mechanisms in carcinogenesis induced by nitrites in NHL.

The present meta-analysis is the largest study to date in which the relationship between nitrate and nitrite consumption from diet or drinking water has been studied in relation to the risk of NHL, including 8,067 NHL cases in total. Based on the studies included in this review, it was possible for us to investigate the relationship in various subgroups using meta-analysis methods. Nevertheless, the meta-analysis had some limitations, due to data originating from previously published observational studies. Firstly, our analysis assembled together published studies in Chinese or English but did not attempt to uncover unpublished data, which may have led to publication bias. However, the Begg’s and Egger’s tests did not suggest any apparent evidence for recall bias. Secondly, in this meta-analysis, the results were principally derived from case-control studies, which could lead to the potential for publication bias, owing to the data for case-control studies being retrospective. Thirdly, due to a number of the subgroup analyses being conducted on tiny datasets, a high degree of confidence should not be placed on their conclusions. Fourthly, although we found a positive relationship between nitrite consumption and DLBCL, we did not obtain sufficient data to compute odds ratios for other particular histopathological subclasses of NHL from the studies included in this meta-analysis. Therefore, we were not able to explore the relationship between nitrate or nitrite consumption and the risk of suffering additional subclasses of NHL. Fifthly, when exploring the relationship between nitrate intake and the risk of NHL, we combined the data for nitrate consumption from diet and drinking water. However, the metrics for nitrate in diet or drinking water was different and we could not find an accurate method for combining them, which would affect the reliability of this meta-analysis. Additionally, a wide range of values for the cutoff points for the highest and lowest level of the consumption of nitrate and nitrite was found in the involved studies, which could led to the possible bias. Finally, the accuracy of the conclusions of this meta-analysis will have been influenced by the threshold values of the highest and lowest categorizations of nitrate or nitrite consumption being distinct in the various studies. Hence, a greater number of well-designed, multi-center, large-sample epidemiological studies are essential for better elucidating the association between the risk of NHL and nitrate or nitrite consumption.

## Conclusion

To summarize, the results suggest that nitrite intake is linked to increased risk of NHL. In the future, to acknowledge our conclusions and to ensure precautions against NHL, additional and more stringent systematic studies are required.

## Materials and Methods

### Literature search

A systematic and comprehensive article retrieval strategy that provided a general impression of the risk of NHL due to nitrate or nitrite consumption was conducted. The Web of Science, the Chinese National Knowledge Infrastructure (CNKI) and PubMed databases were searched for articles of follow-up, cohort or case-control studies assessing the relationship between the risk of NHL and nitrate or nitrite consumption from drinking water or diet, from inception of each database until May 31th, 2019. Few relevant articles were obtained by searching for the terms ‘nitrate’ or ‘nitrite’ and ‘non-Hodgkin lymphoma’. Identification of more relevant articles was accomplished by combing the keywords in a more detailed retrieval strategy, as follow: (N-nitroso compounds OR nitrite OR nitrate) AND (NHL OR non-Hodgkin’s lymphoma OR non-Hodgkin lymphoma OR lymphoma). In addition, a manual search of the references of relevant articles was performed to locate additional studies not identified in the initial search. The current analysis was performed with due consideration to the quality criteria for meta-analyses^44,45^.

### Inclusion and exclusion criteria

All relevant articles were included if they fulfilled the following criteria: (1) a follow-up study or the study had a case-control design; (2) the study investigated the possible relationship between risk of NHL and nitrate or nitrite intake from diet or drinking water; (3) outcomes included relative risk (RR) or odds ratio (OR), or provided sufficient data to perform calculations; (4) the study was published before May 2019 and reported in Chinese or English. If the same samples were reported in different articles, only the manuscript reporting the largest sample size was selected. If an article provided inadequate details or reported overlapping material, it was precluded.

### Data extraction

The following data were retrieved from each article: article title (including publication year and first author’s name), study location of origin, judgment of risk factors, numbers of cases and controls, study design, adjustment factors and patient country of origin. Because non-Hodgkin lymphoma is an uncommon disorder, it is believed that the RR is commensurate with the OR. Thus, OR was adopted to assess any potential association between risk of NHL and nitrate or nitrite consumption. And OR was calculated by comparing the highest level of nitrate or nitrite consumption with the lowest. Two researchers (C.H. and M.X.Y.) independently conducted data collection via a structured questionnaire. A third researcher was consulted in order to reach a consensus, if required.

### Quality assessment

Two investigators independently evaluated the quality of the articles included in the review through application of the nine-star Newcastle-Ottawa Scale (NOS)^46^. In the event of disagreement between the two investigators, a third reviewer arbitrated. The NOS is a tool that permits appraisal of follow-up, population selection, exposure and comparability of included studies. It was then possible to quantitatively assess the quality of each article according to the four aspects above. For each study, the points total varied from 9 to 0. Scores ≥7 represented articles considered high-quality, while scores <7 were considered of low quality.

### Statistical analysis

The relationship between risk of NHL and nitrate or nitrite intake was evaluated using pooled ORs and their 95% confidence intervals (CIs). The choice of random or fixed-effects model was dependent on heterogeneity between studies. Where significant heterogeneity was absent, a Mantel-Haenszel test was utilized to compute pooled ORs in a fixed-effects model^47^. Conversely, the DerSimonian and Laird method was used to evaluate a random-effects model^48^. In order to calculate levels of heterogeneity, the *I*^2^ statistic (values of 0%, >0% and ≤25%, >25% and ≤50%, or >50% implying zero, low, moderate or high heterogeneity, respectively) and the Q test were adopted^48,49^. To detect sources of heterogeneity, we also conducted subgroup analyses, using source of nitrate, study design or quality, geographical area, gender, NHL subtype and levels of nitrate in water. In addition, a Galbraith plot and meta-regression analysis were also performed to explore the potential origins of heterogeneity^50^.

For DR analysis, articles were included if they provided the number of controls and cases in addition to reporting at least 3 levels of nitrate or nitrite intake for every exposure classification. Using a method proposed by Orsini *et al*.^51^ and Greenland *et al*.^52^, we repeated the meta-analysis to re-assess risk. The mean value of every categorization range for nitrate or nitrite intake was used in the DR analysis. If the upper boundary of the highest category or the lower boundary of the lowest category was not provided in a particular study, the equivalent interval was assumed according to the next highest or lowest category^53^. The potential DR relationship of risk of NHL to nitrate or nitrite intake was estimated in two stages^51^. In the first, a limited cubic spline model with 4 knots at the percentages: 5%, 35%, 65% and 95% of the allocated exposure consumption was calculated, then three regression moduli (4 knots minus 1) were computed. Secondly, the covariance from every study was integrated.

In order to assess underlying publication bias, we executed a trim-and-fill test^54^, which indicated the magnitude of influence of all studies allocated normally around the center of a funnel plot. In addition, latent publication bias was also evaluated using an Egger’s test (linear regression method)^55^ and a Begg’s test (rank correlation method)^56^. STATA 11.0 software (StataCorp, College Station, TX) was used to conduct the meta-analysis. *P* values less than 0.05 were considered significant.

## Supplementary information

supplementary information.
