# Supplementary material for: The relationship between consumption of nitrite or nitrate and risk of non-Hodgkin lymphoma
Source: Sci Rep. 2020 Jan 17;10:551. doi: 10.1038/s41598-020-57453-5 (PMC6969097; doi:10.1038/s41598-020-57453-5)
Supplement: Supplementary file 1 — supplementary information. [file 41598_2020_57453_MOESM1_ESM.doc]

**Supplementary information**

**The relationship between consumption of nitrite or nitrate and risk of non-Hodgkin lymphoma**

Mengxia Yu^1, 2*^; Chenying Li^1, 3*^; Chao Hu^1, 3*^; Jingrui Jin^1, 3^; Shenxian Qian^2^; Jie Jin^1, 3^

^1^Department of Hematology, The First Affiliated Hospital, College of Medicine, Zhejiang University, #79 Qingchun Road, Hangzhou, Zhejiang Province, PR China, 310003

^2^Department of Hematology, Hangzhou First People's Hospital, College of Medicine, Zhejiang University, #216 Huansha Road, Hangzhou, Zhejiang Province, PR China, 310006

^3^Key Laboratory of Hematopoietic Malignancies, Diagnosis and Treatment, #17 Laozhedazhi Road, Hangzhou, Zhejiang Province, PR China, 310009

*Mengxia Yu, Chenying Li and Chao Hu contributed equally to this work.

Corresponding author: Prof. Jie Jin (jiej0503@zju.edu.cn)

**Keywords:** non-Hodgkin lymphoma; nitrate; nitrite

**Supplementary Figure**


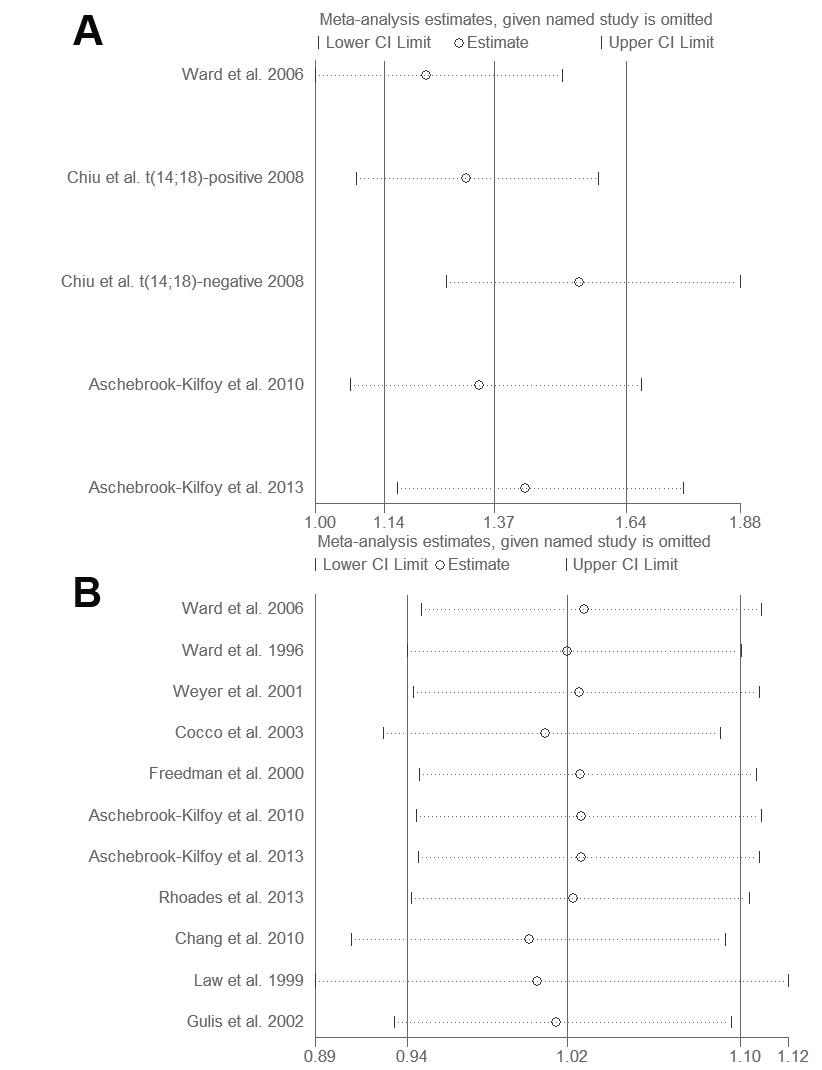


Supplementary Figure S1. Sensitivity analyses by sequential omission of individual studies in the meta-analysis to evaluate heterogeneity in: (A) nitrite and (B) nitrate studies.

**Supplementary Tables**

Table S1. Evaluating the quality of case-control studies and follow-up studies through the nine-star Newcastle-Ottawa Scale.

|  |  | Selecttion |  |  | |  | Comparability | ` |  | Exposure |  | Total stars | |
| --- | --- | --- | --- | --- | --- | --- | --- | --- | --- | --- | --- | --- | --- |
| Study | case definition | representativeness | controls selection | | controls definition |  |  |  | ascertainment | method of ascertainment | response rate | |  |
| Ward^1996^ | 1 | 1 | 1 | | 1 |  | 1 |  | 1 | 1 | 1 | | 8 |
| Ward^2006^ | 1 | 1 | 1 | | 1 |  | 1 |  | 1 | 1 | 0 | | 7 |
| Aschebrook-Kilfoy^2010^ | 1 | 1 | 1 | | 1 |  | 1 |  | 1 | 1 | 0 | | 7 |
| Chiu^2008^ | 1 | 1 | 1 | | 1 |  | 1 |  | 1 | 1 | 1 | | 8 |
| Cocco^2003^ | 1 | 1 | 0 | | 0 |  | 0 |  | 1 | 1 | 1 | | 5 |
| Law^1999^ | 1 | 1 | 0 | | 0 |  | 0 |  | 1 | 1 | 0 | | 4 |
| Freedman^2000^ | 1 | 1 | 1 | | 0 |  | 1 |  | 1 | 1 | 0 | | 6 |
| Gulis^2002^ | 1 | 1 | 0 | | 0 |  | 0 |  | 1 | 1 | 1 | | 5 |
| Aschebrook-Kilfoy^2013^ | 1 | 1 | 0 | | 0 |  | 1 |  | 1 | 1 | 0 | | 5 |
| Chang^2010^ | 1 | 1 | 1 | | 0 |  | 1 |  | 0 | 0 | 1 | | 5 |
| Rhoades^2013^ | 1 | 1 | 1 | | 0 |  | 1 |  | 0 | 1 | 0 | | 5 |

Table S2. Evaluating the quality of cohort study through the nine-star Newcastle-Ottawa Scale.

|  |  | Selecttion |  |  |  | Comparability | ` |  | Exposure |  | Total stars | |
| --- | --- | --- | --- | --- | --- | --- | --- | --- | --- | --- | --- | --- |
| Study | representativeness | the non-exposed cohort | ascertainment | outcome of interest |  |  |  | assessment | follow-up time | adequacy | |  |
| Weyer^2013^ | 1 | 1 | 1 | 0 |  | 1 |  | 1 | 1 | 1 | | 7 |
